# Digital technology and healthcare delivery in insulin-treated adults with diabetes: a proposal for analysis of self-monitoring blood glucose patterns using a dedicated platform

**DOI:** 10.1007/s12020-023-03605-2

**Published:** 2023-11-23

**Authors:** Concetta Irace, Elena Acmet, Antonio Cutruzzolà, Martina Parise, Paola Ponzani, Antonietta Maria Scarpitta, Riccardo Candido

**Affiliations:** 1 Department of Health Science, University Magna Graecia, Catanzaro, Italy; 2 Medical Affairs Director, Roche Diabetes Care, Monza, Italy; 3 Department of Clinical and Experimental Medicine, University Magna Graecia, Catanzaro, Italy; 4 Unit of Diabetology and Metabolism, ASL 4, Chiavari, GE Italy; 5 Unit of Endocrine and Metabolic diseases “Paolo Borsellino” Hospital, Marsala, ASP Trapani Italy; 6 Diabetes Centre, University of Trieste, Azienda Sanitaria Universitaria Giuliano Isontina, Trieste, Italy

**Keywords:** Self-monitoring of blood glucose, Diabetes management, Digital technology, Digital health, Glycemic pattern

## Abstract

**Purpose:**

A remote platform for diabetes care (Roche Diabetes® Care Platform, RDCP) has been developed that allows combined face-to-face consultations and remote patient monitoring (RPM).

**Methods:**

A dedicated flowchart is proposed as a clinical approach to help healthcare professionals in the appropriate interpretation of structured self-monitoring blood glucose data, as visualized on the RDCP during the visits, and in the optimal management of patients using the integrated RDCP-RPM tools.

**Results:**

The platform organizes patterns in different blocks: (i) hypoglycemia; (ii) hyperglycemia; (iii) blood glucose variability; (iv) treatment adherence, which identifies a possible individual pattern according to glycemic control challenges, potential causal factors, and behavioral type patterns. The flowchart proposed for use of the RDCP-RPM is self-explanatory and entails 3 steps: (1) evaluation of quality and quantity of self-monitoring blood glucose data; (2) pattern analysis; (3) personalized suggestions and therapy changes.

**Conclusion:**

The main aim of the remote treatment flowchart proposed is to support healthcare professionals in the identification of hypoglycemic and hyperglycemic patterns using the RDCP regardless of the HbA_1c_ value and ongoing treatment, which however, become crucial in combination with pattern analysis in the therapeutical choice.

## Introduction

Self-monitoring blood glucose (SMBG) is a cornerstone of diabetes management. The utility of SMBG in daily life is evident in insulin-treated patients with type 1 (T1D) and type 2 diabetes (T2D) [1]. It involves regular measurements of blood glucose, allowing patients with diabetes to track glucose levels and make informed decisions before meals, exercise, and other activities. SMBG is a valuable tool helping healthcare providers (HCPs) adjust insulin treatment, improve glucose control, and prevent complications [2]. Despite the recognized benefits of SMBG, roughly 54.6% of patients treated with insulin meet their desired target for glycated hemoglobin (HbA_1c_) [3]. Several patient-related factors contribute to this issue, including inadequate insulin dosing, inappropriate carbohydrate counting, fear of hypoglycemia, and use of an unstructured SMBG [4]. Structured SMBG follows different monitoring patterns and improves glycemic control, [5] variability, and better quality of life in insulin-treated patients [6, 7]. Improvements have recently been reported in the large PRISMA trial wherein structured SMBG showed clinical value in reducing HbA_1c_ in non-insulin-treated T2D, suggesting that this clinical benefit may be mediated by more appropriate and timely changes in drug therapy regardless of the use of insulin [6, 7].

Physician-related barriers may also limit the achievement of good glycemic control in SMBG users, given the difficulty in aggregating and interpreting glucose data efficiently. Integrating a dedicated digital platform in clinical practice could allow HCPs to collect, analyze, and observe data in a user-friendly manner and change treatment both remotely and during a face-to-face visit.

Reviews and meta-analyses have investigated the effectiveness of digital health interventions in improving metabolic control and patient compliance [8–10]. The interventions typically involve mobile medical apps and digital platforms, which leverage the power of connectivity and data analysis to monitor patients and personalize diabetes management.

The COVID-19 pandemic has highlighted the utility of remote patient monitoring (RPM), enabling HCPs to maintain regular contact with patients and manage medications if needed [11]. The RPM approach represents a valid alternative that can ensure continuity of care for individuals with diabetes, which is dramatically increasing worldwide, and providing education and increasing awareness of the disease and its treatment [12, 13].

A remote digital platform for diabetes care (Roche Diabetes® Care Platform, RDCP) has recently been developed that allows combined face-to-face consultations and RPM. The RDCP RPM solution includes a smartphone app (mySugr) that enhances personal diabetes self-management and collects data from glucose meters. The HCPs can carry out RPM using this platform as it has features that track patients, document observations and time spent, send secure and private messages to the patient and receive replies, request a video chat, and directly speak with patients. At the same time, patients can view blood glucose reports, recognize glucose patterns, and notify clinically significant hypoglycemic and hyperglycemic values to the HCPs. The RDCP may be helpful for any patient with type 1, type 2, or gestational diabetes, regardless of the treatment used. In summary, the platform streamlines telemedicine visits, reducing the need for in person apointments. It also supports telemontoring thereby aiding in the identification of patients who may need either an in-person consultation or an extra telemedicine visit, and makes eases the process of in-office traditional visits while also assisting nurses in providing tele-education. The platform has the unicity to connect healthcare professionals and their patients while creating actionable insights to help deliver more efficient personalized care. More than 130 devices can be connected to the RDCP thanks to open connectivity. Importantly, it should be noted that the RDCP complies with all European regulations in terms of privacy and data security and has all the certifications required by national and international regulations. The patient shares data and information with the clinician only through specific consent and can access this information securely, using his or her own account (username and password).

Herein, a dedicated flowchart is proposed to help in the appropriate interpretation of structured SMBG data, as visualized on the RDCP, and in the optimal management of these patients using the integrated RDCP-RPM tools. We also briefly summarize the key points of current guidelines, along with the definition of structured SMBG, and SMBG pattern analysis.

## Guidelines for SMBG regimens

SMBG regimens are classified as intensive and less-intensive based on the number of weekly tests suggested. The choice relies on HbA_1c_, ongoing treatment, risk of hypoglycemia, and the patient’s frailty. Insulin-treated individuals or individuals with HbA_1c_ out of the target, patients on steroid treatment, or concomitant conditions responsible for persistent hyperglycemia deserve an intensive regimen. Conversely, a less intensive regimen is proposed for non-insulin-treated individuals. A number of associations have provided recommendations for SMBG (Table 1).

**Table 1:** Summary of guidelines for SBMG

Society	Key points
American Diabetes Association [14]	SMBG recommended for all patients on insulin regimens to check blood glucose levels:
	• before meals or snacks;
	• at bedtime;
	• before and while performing a critical task;
	• before exercise;
	• when symptoms of hypoglycemia occur;
	• after treating hypoglycemia.
International Diabetes Federation [15]	• Long-term intensive (5 to 7 points: before and after meals and bedtime) SMBG regimen in insulin treated-patients;
	• Short-term intensive (5 to 7-points: before and after meals and bedtime) SMBG regimen recommended over 1–3 days;
	• Sstaggered regimen of 5 to 7 points over 2–3 weeks during infection, stress, traveling, worsening of HbA_1c_, intensification of treatment, pregnancy, or planning to become pregnant in non-insulin-treated T2D.
Diabetes Canada [16]	Different SMBG regimens to be used based upon:
	• the type of diabetes (T1D, T2D, gestational diabetes);
	• HbA_1c_ value (lower or higher than 7%);
	• ongoing treatment (insulin, secretagogue, other drugs, none);
	• presence of concomitant illness;
	• use of steroids;
	• disease duration (less or more than 6 months).

The American Diabetes Association recommends SMBG for all patients on insulin regimens to check blood glucose levels before meals or snacks, at bedtime, before and while performing a critical task (e.g.,—as this could include other activities beyond driving), before exercise, when symptoms of hypoglycemia occur, and after treating hypoglycemia [1]. SMBG promotes insulin titration and correction of hyper- and hypoglycemia [14].

The International Diabetes Federation guidelines on SMBG suggest, beyond the consolidated strategy in insulin-treated patients, a short-term intensive (5 to 7-points: before and after meals and bedtime) SMBG regimen over 1–3 days or staggered regimen of 5 to 7 points over 2–3 weeks during infection, stress, traveling, worsening of HbA_1c_, intensification of treatment, pregnancy, or planning to become pregnant in non-insulin-treated T2D as a valid strategy for making a therapeutic decision and implementing lifestyle modification [15].

Diabetes Canada proposes different SMBG regimens based upon the type of diabetes (T1D, T2D, gestational diabetes), HbA_1c_ value (lower or higher than 7%), ongoing treatment (insulin, secretagogue, other drugs, none), presence of concomitant illness, use of steroids, and disease duration (less or more than 6 months) [16]. For example, a 3-7-point profile should be regularly obtained by patients using insulin more than once daily; a 7-point profile every 1–3 months in individuals not receiving insulin and with HbA_1c_ not at the target; 4 times per day, including overnight when hypoglycemic episodes occur.

In any case, personalized strategies can be suggested in case of illness, steroid treatment, surgery, or any other clinically relevant condition. As an additional consideration, SMBG can support more treatment decisions when glycated hemoglobin is unreliable (e.g., anemia, pregnancy, chronic kidney disease, etc.) or anomalous readings are obtained.

## Structured SMBG

**Fig. 1:**
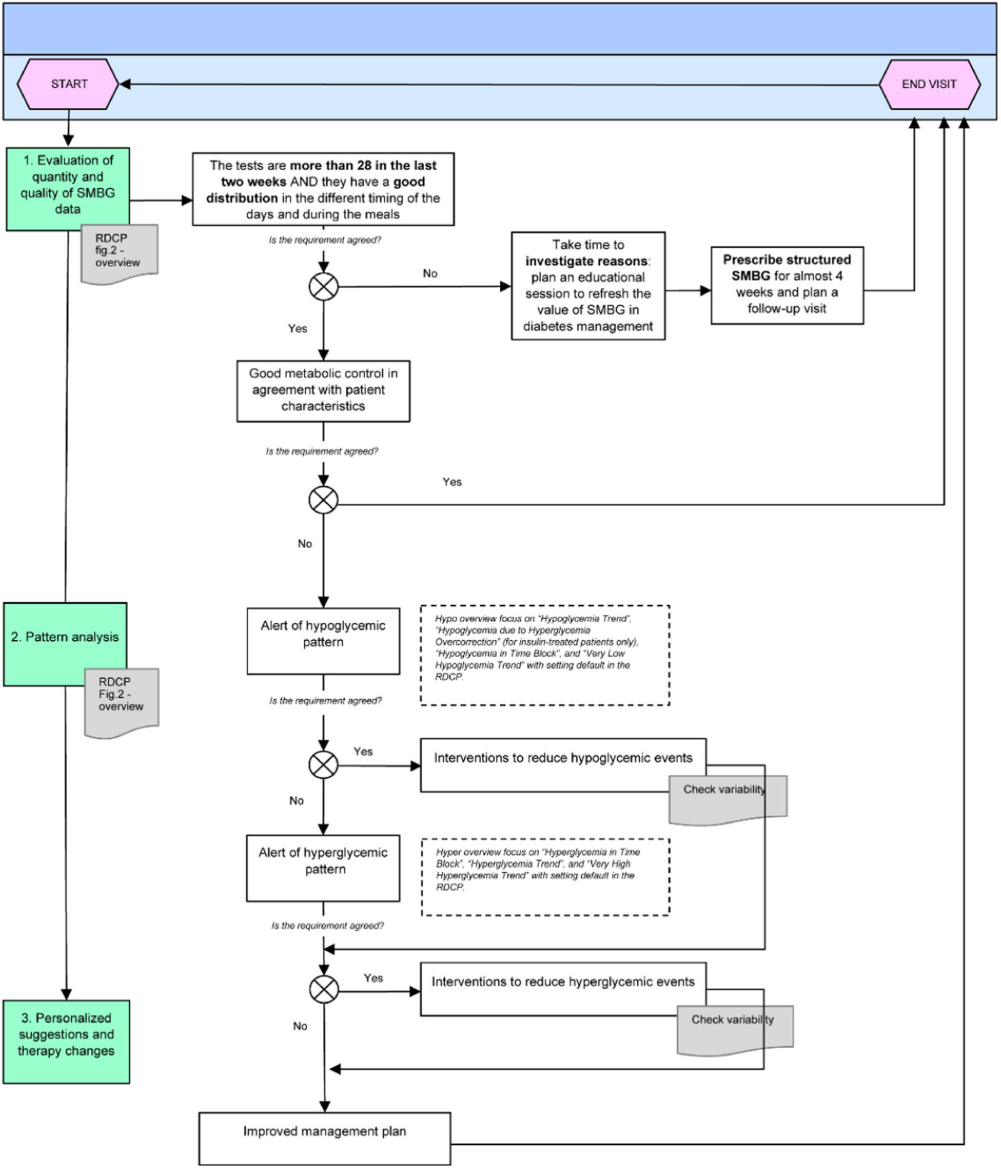
Diabetes management flowchart with SMBG data in T2D using the RDCP with pattern analysis

The term structured SMBG includes the timing of glucose control and the actions taken based on glycemic values. Structured SMBG must be included in a more comprehensive program, including diabetes education, understanding, and behavioral changes [15]. SMBG has been evaluated in clinical trials for insulin and non-insulin-treated patients. The STeP study, a 12-month prospective study, assessed the efficacy of structured SMBG testing in insulin-treated T2D patients. The structured testing included a 7-point profile (fasting, pre-prandial, postprandial, and bedtime) for 3 consecutive days prior to the scheduled visit (Fig. 1S) [17]. The medical staff was trained in interpreting structured data and changing ongoing treatment according to an algorithm that described various pharmacologic and lifestyle treatment strategies in response to specific SMBG patterns. The 4-step training program consisted of Step 1, Identification and prioritization of the glycemic abnormality (Priority 1 – Hypoglycemia; Priority 2 – Fasting Hyperglycemia; Priority 3 – Postprandial Hyperglycemia); Step 2, Identification of the timing and frequency of glycemic abnormalities; Step 3, Investigation of potential causes; Step 4, Take action. Structured SMBG was associated with a significant decrease in HbA_1c_ (−1.2%) compared to non-structured blood glucose testing [17].

The PRISMA Study, carried out in Italy, demonstrated the reduction of HbA_1c_ by structured SMBG combined with a dashboard illustrating mean pre- and post-meal blood glucose and magnitude of postprandial glucose excursion over 4 consecutive weeks in non-insulin-treated patients with type 2 diabetes [18].

## SMBG and pattern analysis

The widespread use of graphical representation of SMBG data has prompted experts to characterize blood glucose data using pattern analysis despite calculating averages [19]. A blood glucose pattern can be defined as a series of high or low values at the same time of the day on different days. Currently, there is no consensus about the number of repeated high and low values characterizing a pattern. However, recent clinical research evaluated the performance of a glucose pattern-recognizing tool incorporated in the SMBG system that defined the recurrence of a series of 3 values above the target as a hyperglycemic pattern and as a series of 2 values below the target as a hypoglycemic pattern [20].

The pattern analysis involves retrospective SMBG data analyses and must be placed in an evaluation setting, including carbohydrate intake, medication administration (type, dosage, timing), physical activity, sedentary lifestyle, and stress. The comprehensive analysis of glucose patterns allows healthcare professionals to implement appropriate decisions and propose targeted therapeutic changes [21, 22]. The pattern management approach includes different steps: (i) establish pre-meal and post-meal blood glucose targets; (ii) analyze data to determine whether any patterns emerge; (iii) assess if any influencing factor exists; (iv) take action; (v) regularly monitor SMBG to evaluate the impact of the action taken.

Patients can be educated to approach pattern analysis, evaluate how well-controlled their glucose value is, and self-promote therapeutic changes to minimize the high and low glucose values, contributing to more stable glucose levels.

Notably, interday variability can affect pattern analyses, making treatment change difficult. If high variability exists, SMBG should be intensified before analyzing the pattern and changing treatment. Common measures of glycemic variability include standard deviation (SD), low blood glucose index (LBGI), high blood glucose index (HBGI), and coefficient of variation (CV) [23–26]. However, physicians are also familiar with SD, and the International Consensus on Time in Range has proposed the evaluation of interday variability with the CV of mean daily glucose and suggests the threshold of ≤36%. Some studies suggest that a CV < 33% can provide additional protection against hypoglycemia during treatment with insulin or sulfonylureas [27]. In conclusion, pattern analysis offers an exceptional opportunity to identify problems, solve them together, and address diabetes management challenges.

## Flowchart for the use of RDCP pattern analysis

The RDCP uses a more comprehensive pattern management approach by overcoming the standard definition of hypo- and hyperglycemic patterns. The platform organizes patterns in 4 different blocks: (i) hypoglycemia; (ii) hyperglycemia; (iii) blood glucose variability; (iv) treatment adherence, which identifies a possible individual pattern according to glycemic control challenges, potential causal factors and behavioral type patterns (Table 2). Due to reasons of accuracy, the pattern analysis is activated if the minimum number of tests are performed in 4 weeks (default 72 tests, settable range 30–120). The frequency and the time of blood glucose measurements are suggested by physicians according to guidelines and the patient’s characteristics.

**Table 2:** Individual blood glucose patterns according to pattern blocks

Pattern Block	Individual Patterns
Hypoglycemia	Hypoglycemic trend
	Hypoglycemia due to hyperglycemia overcorrection
	Hypoglycemia frequency in time block
	Hypoglycemia repetition in days
Hyperglycemia	Hyperglycemic trend
	Hyperglycemia due to overcorrection of hypoglycemia
	Hyperglycemia frequency in time block
	Hyperglycemia repetition in days
	Hyperglycemia due to missed intake bolus
Variability	Standard deviation
	Low blood glucose index (LBGI)
	High blood glucose index (HBGI)
Treatment adherence	Frequency of blood glucose tests

The patterns offer an exceptional opportunity for collaboration between HCPs and people with diabetes to identify, solve problems together and address diabetes management. The design is intended to alert to a pattern and provide visualization of the glucose tests that created the patterns. This strategy can enhance patient awareness of personal glucose control and self-learning, facilitating communication and shared decision-making about diabetes self-management goals.

Thus, pattern analysis can be considered an evolution of traditional SMBG markers of hyper- and hypoglycemia (HBGI, LBGI). We herein propose a simple flowchart approaching the RDCP pattern analysis for patients on an insulin regimen aimed to implement education, insulin titration, and optimization of treatment at the onset of diabetes. In good, stable metabolic control, no pattern analysis is suggested unless potentially glucose-worsening events occur (infection, stress, pregnancy, planning pregnancy, steroid treatment). The flowchart is self-explanatory and entails 3 steps (Fig. 1).

### Step 1. Evaluation of quality and quantity of SMBG data

**Fig. 2:**
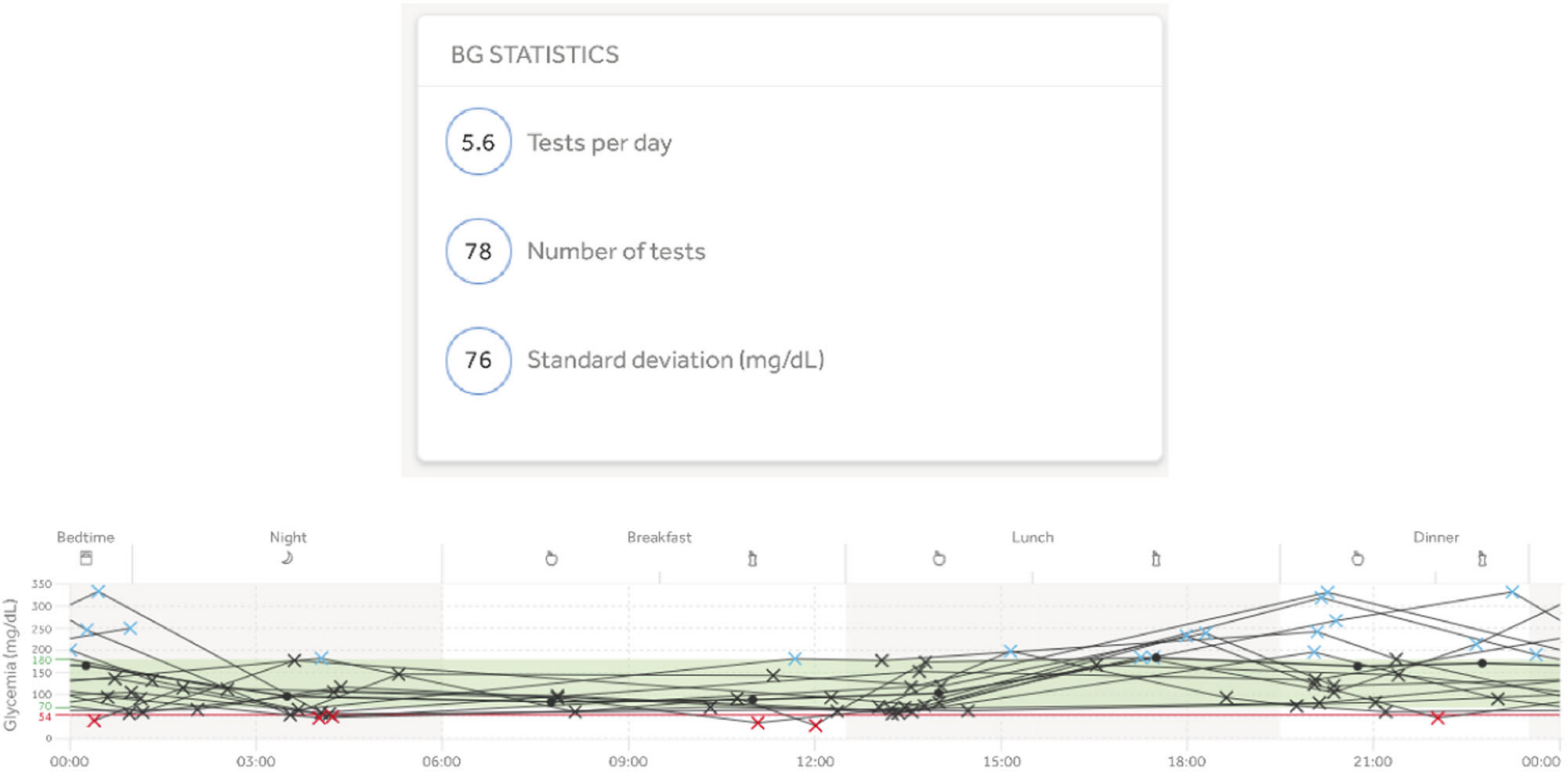
Example to evaluate the quantity and quality of SMBG data using the RDCP

The flowchart begins with assessing the quantity (suggested minimum 28 tests in 2 weeks) and quality of SMBG data, according to guidelines and clinical judgment. The quality of SMBG data refers to the recommended testing time (morning, pre-meal, post-meal, pre-exercise, etc.). An example of the evaluation of the quantity and quality of SMBG data is shown in Fig. 2, which illustrates the mean number of tests per day and the distribution of blood glucose tests during the day.

If the quality and quantity of tests are not acceptable, the reasons for low adherence to SMBG prescription should be investigated. An educational session should be planned to help patients better understand the value of SMBG in diabetes management. However, before planning a new visit, even remotely, critical issues (single hypoglycemic and hyperglycemic events) should be examined and discussed.

### Step 2. Pattern analysis

**Fig. 3:**
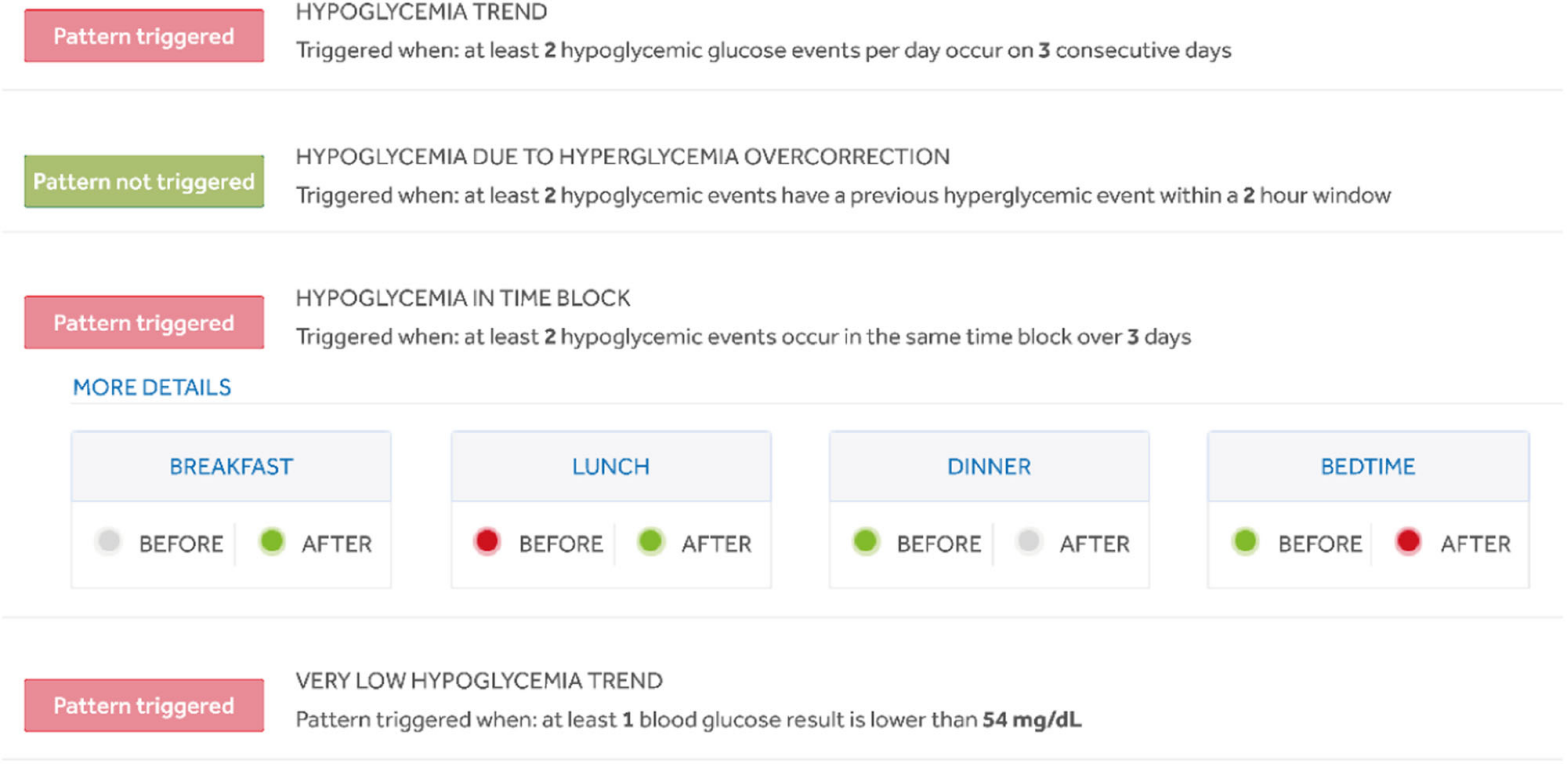
Example of defining the hypoglycemic pattern and illustrating the pattern using the RDCP

Before analyzing the glycemic pattern, the number of blood glucose tests needed to identify each pattern must be set according to patient characteristics (i.e., frailty, pregnancy, high hypoglycemic risk, hypoglycemia unawareness) and ongoing treatment. In Table 3 we describe the clinical use, how the platform works, and the settable ranges by different patterns. The platform identifies patterns occurring before and after meals, bedtime, and nighttime (red light). An example is illustrated in Fig. 3. Graphs and daily registry showing the episodes are also available to be commented on with the persons with diabetes for educational purposes and to contextualize any single event (Fig. 2S).

**Table 3:** Definition and mode of operation of the RDCP patterns

Pattern	Clinical use	How the platform works	Settable ranges
Hyperglycemia Trend	It helps to detect hyperglycemic events occurring on consecutive days	An indicator turns red when there are 2 or more glucose tests in the hyperglycemic range per day on 3 consecutive days	Glucose tests in the hyperglycemic range per day: 1–5 Consecutive days:1–10
Very High Hyperglycemia Trend	It helps to detect the frequency of very high hyperglycemic events	An indicator turns red when there are 1 or more glucose tests that are above 250 mg/dL	Glucose tests: 1–10 Glucose levels: 140–300 mg/dL
Hypoglycemia Trend	It helps to detect hypoglycemic events occurring on consecutive days	An indicator turns red when there are 1 or more hypoglycemic glucose events per day on 2 consecutive days	Hypoglycemic glucose events per day: 1–5 Consecutive days: 2–10
Hypoglycemia in Time Block	It helps to detect hypoglycemic events occurring in the same time block	An indicator turns red when 2 or more hypoglycemia events happen in same time block over 7 days during the analysis time period	Hypoglycemia events in same time block: 2–10 Days: 2–10
Very Low Hypoglycemia Trend	It helps to detect the frequency of very low hypoglycemic events	An indicator turns red when there are 1 or more glucose tests below 54 mg/dL in the analysis time period	Glucose tests: 1–10 Glucose levels: 50–80 mg/dL

### Step 3. Personalized suggestions and therapy changes

The last step of the flowchart involves making individualized therapeutic changes according to RDCP pattern analysis to reduce the burden of hypo- and hyperglycemia. For example, HCP could suggest intensifying or de-intensify ongoing treatment, measuring blood sugar more frequently, having a balanced diet, avoiding skipping meals, exercising regularly, taking medication as prescribed, investigating attitudes that can increase the risk of hypo- and hyperglycemia, and reinforcing the rules to correct hypo- and hyperglycemia. The insulin sensitivity factor and the carbohydrate-to-insulin ratio can also be revised if necessary. Finally, to improve the overall management plan, it might be suggested to insert notes on diet and physical activity using a dedicated app to improve self-education and management. The effect of treatment changes can be quickly evaluated thanks to the opportunity that the platform offers to compare time intervals.

Before changing ongoing treatment, we suggest evaluating glycemic variability. Indeed, in case of high variability, the intensification or de-intensification of therapy may increase the risk of hypoglycemia or hyperglycemia. The RDCP also allows the evaluation of variability by SD, CV, LBGI, and HBGI, which are available in the blood glucose report.

## Conclusions

The RDCP, along with glucose pattern management, can be used to support HCPs to determine therapeutic changes needed and aid people with diabetes to be more adherent to SMBG and understand how to optimize blood glucose control in daily life. While continuous glucose monitoring is replacing the traditional meter in insulin treatment, SMBG is still largely used in non-insulin treated patients with T2D or by patients undergoing basal oral/incretin therapy. The 3-step flow chart proposed summarizes the potentiality of the RDCP in organizing, analyzing, and interpreting glucose data. In particular, hypoglycemic and hyperglycemic pattern analysis is the mainstream of a patient-centered diabetes management approach. The number and the rate of glucose testing of each pattern can be personalized according to patients’ needs and characteristics. Furthermore, the integration with glycemic variability allows HCPs to make appropriate decisions without increasing the risk of hypoglycemia.

The RDCP helps HCPs during face-to-face visits and is crucial during remote monitoring. Indeed, patients with diabetes need continuity of care, periodic self-management training, and treatment adjustment, which can be limited by the long-distance to the center for the care of diabetes, geographical barriers, and waiting lists.

All learnings of the past few years are focused on the best use of available digital technologies in order to guarantee therapeutic continuity and support persons with diabetes and their families, also remotely and in difficult situations. Evidence including different platforms for the remote upload and analyses of glucose data, has highlighted the role of diabetes technologies as a safe and effective tool in managing diabetes and improving the quality of life [11, 28–30]. In conclusion, the RDCP and the proposed flow chart offer the opportunity to facilitate the application and interpretation of glucose data according to current SMBG Guidelines.

### Supplementary Information

Supplementary Information
