# Supplementary material for: Digital technology and healthcare delivery in insulin-treated adults with diabetes: a proposal for analysis of self-monitoring blood glucose patterns using a dedicated platform
Source: Endocrine. 2023 Nov 23;84(2):441–9. doi: 10.1007/s12020-023-03605-2 (PMC11076319; doi:10.1007/s12020-023-03605-2)

Figure 1S. Example of a 7-point SMBG profile tool.


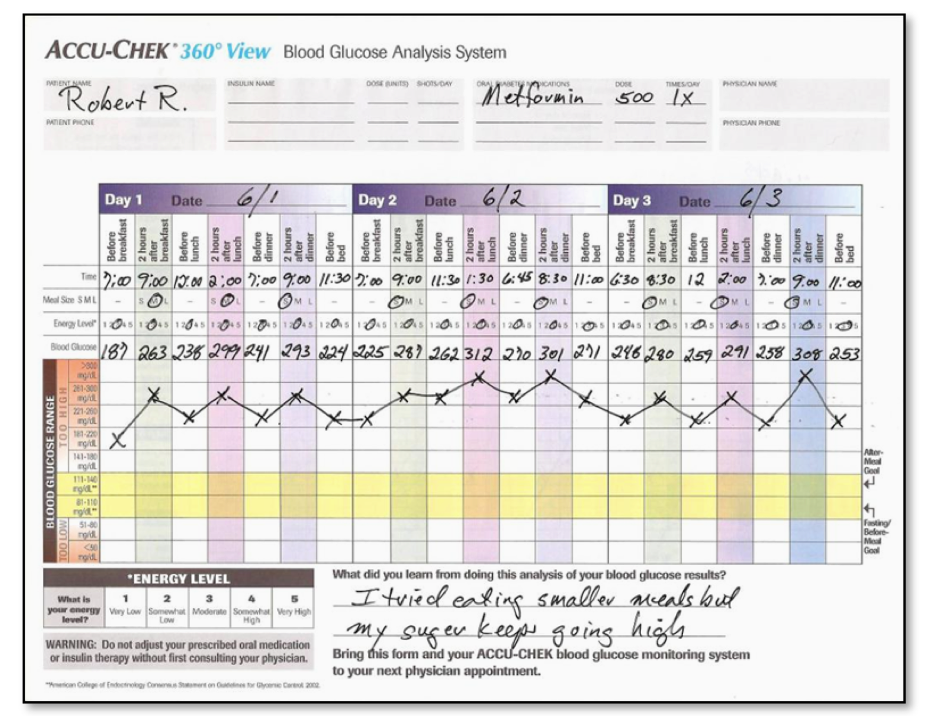


Figure 2S: Graph and registry illustrating hypoglycemic events.


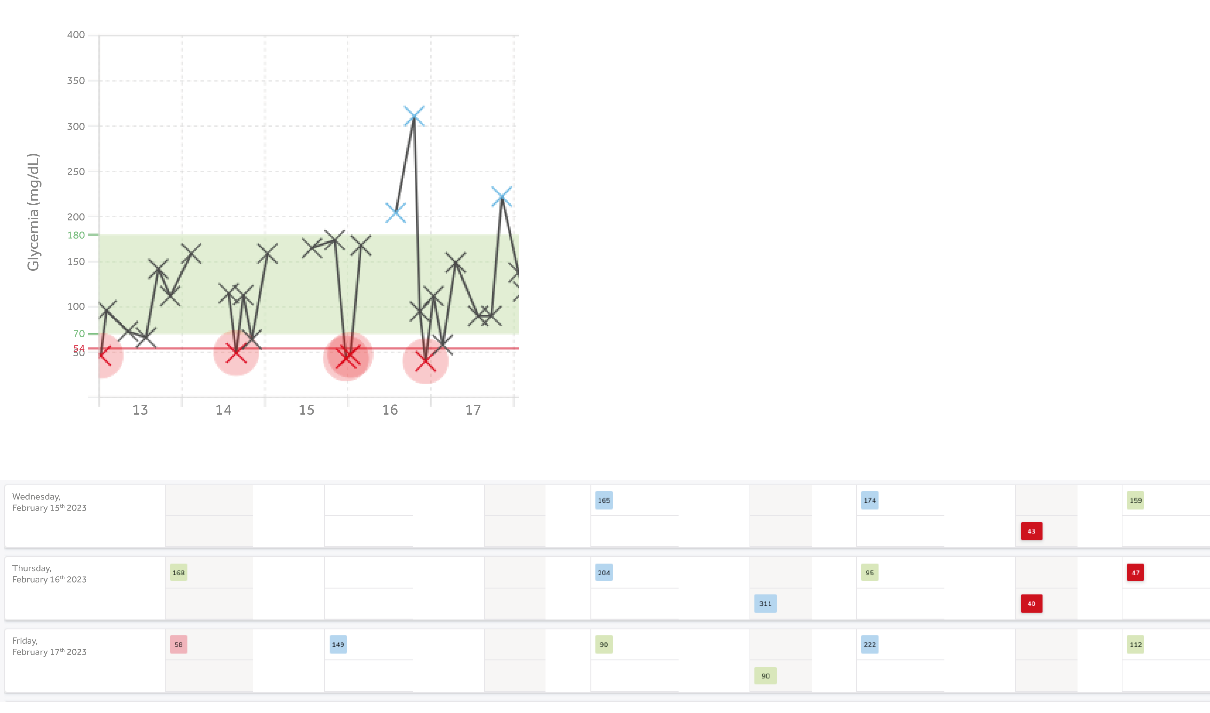

Supplement: Supplementary file 1 — Supplementary Information [file 12020_2023_3605_MOESM1_ESM.docx]
